# Understanding the public stigma of mental illness: a mixed-methods, multi-level, exploratory triangulation study

**DOI:** 10.1186/s40359-024-01887-3

**Published:** 2024-07-20

**Authors:** Daniel Walsh, Juliet Foster

**Affiliations:** 1 Grenoble Alpes University, Bureau 49 Sciences Po/ Pacte, Grenoble, 38040 France; 2 King’s College London, 16 De Crespigny Park, London, SE5 8AB UK

**Keywords:** Public understanding, Stigma, Mental Illness, Social Contact, Themata, Public Health, Unintended Consequences

## Abstract

**Background:**

This study examines the role of themata in understanding mental health-related stigma. It is motivated by the need for alternative theoretical-methodological approaches beyond the dominant frameworks in education and contact-based anti-stigma public health efforts, which have shown mixed effects. Specifically, it addresses the need for a more nuanced framework in stigma research, one that is sensitive to the dialogues through which people relate themselves to mental health and stigma in context.

**Methods:**

The research employs an exploratory mixed-methods approach, including the analysis of 529 news reports, 20 focus group discussions, and 19 one-to-one interviews, all concerning representations of shared living arrangements with someone perceived to have experiences of mental illness. Thematic analysis and natural language processing are used within a convergent triangulation design to analyze the data.

**Results:**

We found that mental health and illness were communicated through an overarching Self/Other thema and five subordinate themata: normal/abnormal, harm/non-harm, bounded/non-bounded, and moral/immoral. Despite familiarity with psychological distress and ‘modern’ explanations of mental illness, concerns about social identity motivated representations of mental illness as a predominantly permanent, negative form of personhood marked by abnormality, harm, distance, and immorality. Additionally, concerns about personal vulnerability, including historically rooted fears of contagion, motivated distancing representations of mental illness, rather than neutral portrayals.

**Conclusions:**

Themata have under-developed theoretical and methodological potential for addressing mental health-related stigma, particularly in their ability to describe the dynamic ways in which culture motivates people to both resist and reproduce stigma, partly through ambivalences, absences, tensions, and ambiguities in representation. A critical discussion is provided on how themata may support ecological strategies in mental health campaigns over generic models, emphasizing the need to understand group knowledge and contact dynamics to mitigate adverse effects. Themata Public Health Unintended Consequences Mixed Methods Behaviour Change Natural Language Processing.

## Background

### Introduction

A new approach to mental health-related stigma is needed because current dominant strategies do not adequately address the public’s desire to avoid intimate or sensitive forms of contact (e.g., in the home) with individuals with experiences of mental illness. This is despite increases in opinions concerning the unacceptability of stigma and greater familiarity with professional psychiatric terminology (e.g., depression, schizophrenia) and biomedical explanations (e.g., genetics, neurochemicals) [1]. This is a serious issue, as current models for alleviating mental health-related stigma assume that increasing public knowledge will linearly improve attitudes toward mental illness and reduce social distance [1]. Education and contact are the dominant strategies used to address public stigma [2–5]. These approaches aim to address the long-standing public perception that mental illness is ‘unfamiliar’ or ‘unknown’ [1]. However, education-focused strategies, particularly those that emphasize a bio-genetic basis for mental illness, unfortunately correlate with increased desires for social distance [3, 6]. Social distance is a dynamic concept that refers to the felt sense of affinity or dissonance between perceived groups. It is often communicated through descriptions of the perceived non-familiarity and incomprehensibility of experiences of mental illness [1, 3]. Similarly, contact-based interventions often have mixed or no effects [7]. This may be due to the complex mix of positive and negative experiences people have with ‘contact’ related to mental ill-health, as well as the various ways contact can occur with representations of mental illness (e.g., personal thoughts, conversations with friends and family, media) [1, 8].

In this paper, we present an alternative framework for conceptualizing stigma, which centers on representation and communication. ‘Mental illness’ is communicated in society through various forms, from media portrayals and conversations with friends to personal reflections [1, 8]. These communications are not neutral or consistent [1]; they are replete with feelings and opinions [1, 8]. To explain ‘mental illness’ to ourselves and others, we draw on shared understandings. For example, while we might not agree with certainty about what constitutes contact with mental illness, we can oppose the perceived ‘harms’ of contact and the perceived ‘safety’ of no contact. From this perspective, we can think about mental illness in terms of dialogues grounded in shared oppositions or themata (e.g., Self/Other; Harm/Safe; Pure/Impure) that societies employ to maintain, develop, and challenge representations of social issues [9, 10].

Themata are dialogical units with an oppositional structure [9]. Studies of everyday thinking find that people rarely hold complete or secure beliefs about health and illness [8]. Instead, they offer partial explanations, often referring to stories of their own experiences or those of friends and family. In these explanations, people often argue against themselves or leave them partial or open-ended [1]. These dialogues are underpinned by taken-for-granted oppositions latent in communication, such as comparisons of the perceived relative ‘normality’ of experiences of sadness or anxiety compared to the ‘abnormalities’ presented by perceived mental illnesses such as ‘schizophrenia’ [1, 4, 5]. In other words, we understand ourselves in relation to mental illness by opposing ‘normal’ with ‘abnormal’ [8]. These oppositions are dynamic, meaning that what is accepted as ‘normal’ in one social context (e.g., shared public spaces) may be perceived differently in ‘private’ spaces [1].

This emphasis on themata could pave the way for potential advancements in public anti-stigma efforts: identifying these relational units of communication allows for an exploration of the dynamic manners in which individuals make sense of mental health-related stigma in various contexts. For instance, a review of three decades of population research suggests that the degree of pro-social feelings (e.g., the felt need to help) and anti-social feelings (e.g., fear, anger) varies with the perceived differences in forms of social contact (e.g., subtenant vs. neighbour) as well as the presence and ‘type’ of diagnostic label (e.g., schizophrenia vs. depression), in comparison to non-labelled symptoms [11]. Furthermore, identifying themata might offer insights into the motivated aspects of stigma. This approach helps us understand how people represent ‘mental illness’ (for example, seeing it as dangerous) and the motivations underpinning why they have these representations (such as thinking that mental illness is something that happens to others, not themselves). National studies consistently find strong negative reactions to close contact with people with mental illness, especially in intimate settings like the home, even though stigmatizing them is recognized as wrong [12]. These negative views often draw on longstanding stereotypes, such as associating ‘madness’ with violence [13, 14].

In the subsequent sections, drawing on the literature concerning mental health-related anti-stigma programs, we will discuss the challenges in addressing the public’s motivations that perpetuate mental health-related stigma. We will also further elucidate the benefits of emphasizing themata over conventional methods for social research in health and stigma, and the opportunities for drawing on advances in natural language processing to draw out the latent aspects of communication.

### Mental health-related anti-stigma campaigns

Over the last decade, concern over the limited and unintended consequences of anti-stigma efforts has focused attention on the assumptions underpinning mental health-related anti-stigma campaigns [1, 3, 4]. Predominantly, efforts to challenge public stigma—"the contextual climate of prejudice and discrimination" ([14], p. 94)—employ a deficit model of public understanding [15]. This model conceptualizes lay understanding as largely derived from professional knowledge [1, 16].

Practitioners primarily employ educational strategies to remediate this perceived deficit, often combined with elements of social contact, especially in high-income countries [1, 2, 6, 17]. For a discussion of variability in change strategies, see [1]. In line with the deficit model, practitioners hope that promoting professionalized knowledge to the public will translate into positive attitudes and behaviours towards individuals with experiences of mental illness [1, 16]. Unfortunately, despite evidence that interventions improve positive attitudes in the short term and that these attitudinal changes may be attenuated, it is unlikely that current interventions challenge the contact taboos foundational to the reproduction of stigma in society [9, 18–23]. For example, an ethnographic study found that families hosting a lodger with a mental illness, despite being in regular contact with psychiatric professionals for advice on managing mental illness, maintained several rituals to avoid intimate contact, such as separating eating utensils [18]. A contact taboo is a rule that forbids certain actions toward perceived out-groups because they are believed to be either too sacred or too dangerous for ‘normal’ people [18]. Contact taboos frequently encompass implicit beliefs regarding the risks (to the in-group) associated with proximity to mental illness. For instance, in the British media, although the Mental Health Bill of 2002 was broadly portrayed as unduly restrictive—given its potential to mandate compulsory treatment and detainment under an expanded definition of ‘mental disorder’—there was also an implication that individuals with a history of mental ill-health are predisposed to criminal violence likely reproducing the public’s felt need for social distance [16].

### Mental Illness as other

Anti-stigma efforts have done little to challenge, and may have even sustained, public representations of mental illness as "Other" [1, 3, 24, 25]. To "Other" means to represent an out-group as profoundly and undesirably different from oneself and one’s in-group [26]. While it was hoped that by making incremental improvements to dominant change strategies, such as a greater focus on group or curriculum, might displace a representation of mental illness as "Other", public motivations to "Other" go beyond what's considered in generic change programs. Indeed, "Othering" likely constitutes a ‘wicked problem’ ([4], p. 1158) and is sustained by power inequalities in the social order [1, 3]. When close attention is paid to linguistic practices, such as symbol, metaphor, and imagery, findings suggest "Othering" profoundly constrains ways of understanding mental illness, even through anti-stigma campaigns [16, 27, 28].

Our study is derived in response to the ineffectiveness of public mental health anti-stigma campaigns in England, particularly the ongoing tendency of the public to "Other" mental illness. Time to Change (TTC), the leading anti-stigma campaign from 2007 to 2021, was associated with moderate increases in professional knowledge, positive attitudes, and slight decreases in a desire for social distance [29]. However, "Othering" remained prevalent [25, 27, 30]. Public motivations to "Other" experiences of mental illness meant the content promoted through the TTC campaign, especially in its first phase (2007 – 2011), did not fundamentally change public understanding but likely refashioned stigma [16]. Initially, social marketing campaigns emphasized a biomedical and neurogenetic basis for mental illness, often in the form of ‘myth busting’ [3, 16]. Campaigners hoped that increases in professional knowledge would decrease public associations between responsibility and mental illness [3, 16]. Yet, the emphasis on bio-genetic and neurochemical causes did little to challenge the divisive ‘us vs them’ narrative [3, 16, 30]. It also likely reinforced an association between mental illness and permanence [1, 24].

To address contact fears, the later stages of the TTC campaign increasingly used para-social contact, such as stories of empathy and humor in friendships between individuals with and without potential experiences of mental ill-health [29, 31]. However, instead of displacing stigma, "Othering" was likely latent and nuanced [1, 30, 60]. Although there was a notable reduction of ‘bad news’ tweets from the UK national press, especially regarding service provision, "Othering" was likely perpetuated through the continued use of sensationalist imagery [27] and differentiation in public sympathy, perceptions of dangerousness, and unpredictability related to mood and psychosis ([1, 14, 32], 2020 edition). Stories of criminal cases involving individuals with experiences of mental illness, especially schizophrenia, continued to be over-represented in the British media [16, 27]. These narratives likely affirmed public beliefs of dangers associated with mental illness, and through their regular emotive context, bias lay thinking towards violence over mundane negotiations of mental health and illness in everyday life [16, 27]. Moreover, the notion that the public lacked ‘contact’ was misleading, especially since public experiences of psychological distress are widespread [1]. Indeed, this approach further differentiated representations of what one or those similar could experience (e.g., feelings of sadness) from the perceived "Otherness" of mental illness [1, 30].

### Themes and themata

Practitioners may benefit from increased sensitivity to the public’s motivated ways of understanding mental health and illness, especially considering their robust capacity to “Other” [29]. However, prevailing methods for describing public comprehension do not adequately capture how people maintain mental health-related stigma in their daily lives [33]. In this paper, we propose themata—a concept developed in the history of science by Gerald Holton [9]—as a methodological innovation better suited for understanding how people relate to mental health-related stigma. We also explore the possibilities of using natural language processing to address the subtle linguistic features of representation.

In contrast to regular operations of public mental health research, which focus on evaluating variance in discrete and largely fixed components of public understandings of mental illness (e.g., attitudes, knowledge, belief), themata acknowledge that in natural communication, people regularly argue against themselves regarding what constitutes ‘mental illness’ and the perceived risks it poses to them [1]. This is important because by focusing on units through which representations are ambiguously developed and contested (e.g., familiar/non-familiar; moral/immoral), we have a framework to consider the reasons why we see inconsistencies in public behaviour towards mental health-related stigma [1]. For instance, it is a recurring finding that mental illness is perceived as foreign, unknown, or unfamiliar. Yet, this perception often coincides with the acknowledgment of the familiarity of depression, which serves to affirm the perceived fundamental incomprehensibility of schizophrenia, thereby reinforcing a unified representation of mental illness as ‘Other’ [34]. Similarly, while progressive narratives of mental health stigma frequently include discussions of mental health challenges faced by close friends and family members, this is often done to delineate a ‘good’ type of mental illness that is acceptable to be associated with, thereby reinforcing the desire to distance oneself from a ‘bad’ or Othered form of mental illness [35].

Clustering algorithms, as part of natural language processing (NLP), offer the opportunity to highlight the words around which other words cluster [33]. This is an important advancement, as in the regular practice of identifying repeated patterns, as is common in thematic analysis, we can overlook the subtle but important ways people relate to mental illness, relying on what is more easily recognizable to us as researchers, such as discrete labels of diagnoses (e.g., schizophrenia), emotions (e.g., fear), and causes (e.g., genetics) [33]. Yet, focused readings of the text can reveal overlooked public uncertainties and concerns about mental illness, such as metaphors that represent mental illness as a ‘maze’ or images that represent mental illness as ‘dirty’ [27, 32, 33]. Clustering algorithms can be helpful in spotlighting the words around which other words cluster, suggesting latent meanings in the text [33].

In this paper, we pay special attention to the communications between Self and Other, which has been proposed as an epistemological thema in representations of risk and social identity [36, 37]. We understand social identity as the "processes of interpersonal communication" ([38], p.2), which influence the structure and content of social categorization [9, 39]. A Self/Other thema has been found to organize lay representations of various perceived risks in health and stigma, including communicable diseases, organ donation and transplantation, and mental health and illness [15, 37, 40, 41]. In all these cases, the perceived risk is linked to the Self, instinctively shaping certain ways of 'knowing' the object of concern [9, 10]. Specifically, the perceived risk is associated with the affected marginalized out-group (e.g., HIV/AIDS and gay men), limiting alternative forms of social identity [15, 37, 40]. Moreover, the implicit dialogues between Self and Other are inclined to blame out-groups for their marginalization, perpetuating societal stigma [15, 37, 40]. The emphasis on a Self/Other thema aligns with the concept of discourse in their shared rejection of the idea that people have a fixed, asocial self [9]. Instead, it is through our relations with society that we understand ourselves as capable of psychological experience [9]. However, the two are not synonymous in relation to power, especially regarding opportunities for minority groups to express agency and creativity in representation beyond self-regulatory processes, which hold the potential to reshape themselves and wider society [9].

A theoretical-methodological concern advanced in this introductory section has been the challenges in researching why people may disavow stigma and draw on professional knowledge to explain it, but maintain subtle prohibitions on intimate forms of contact, such as within the home or with perceived vulnerable groups [1]. For this reason, we use the context of students’ shared living arrangements. We explore how, in this perceived contact, people formulate representations of themselves in relation to the perceived Otherness of mental illness, a practice that may include comparing mental illness diagnostic labels (e.g., schizophrenia vs. depression) [18, 33].

### Interim summary and contributions

Dominant educational and contact-based interventions aimed at addressing mental health-related stigma are found to yield limited and unintended outcomes. We propose that themata can function as a theoretical-methodological framework to overcome significant limitations inherent in current anti-stigma efforts. Specifically, they offer a means to articulate the motivated ambivalences in contact and knowledge that perpetuate stigma within society, in particular the latent perceived risks posed to the Self posed by contact with the ‘Otherness’ of mental illness. In the remainder of this paper, we will present an empirical example illustrating the themata through which mental health-related stigma is maintained in the context of students’ shared living situations.

We will outline three principal contributions to the literature. First, we will demonstrate how a natural language clustering algorithm can reveal potential latent meanings within text. Specifically, while confirming established findings that public apprehension about social contact is driven by fears of harm, our application of natural language processing suggests that these concerns may also include subtle fears of harm through contagion, a concept historically linked with madness [3, 18]. Second, our novel methodological approach will identify key empirical contributions, notably in pinpointing absences and ambivalences in representation. This encompasses the public’s ambivalent concerns about social contact. These concerns arise from both perceived non-familiarity and similarities to experiences deemed relatable to the Self, along with the underlying belief that the risk associated with mental illness is enduring. Third, the relational analysis of communication, facilitated by the identification of themata, allows for a nuanced development of alternative anti-stigma strategies. Specifically, as our analysis will demonstrate the interdependencies between themata, such as Self/Other and harm/non-harm, we will critically examine how efforts to change public understanding through one-dimensional interventions (e.g., education that individuals with mental illness are not inherently violent) fail to address the negative emotions (e.g., fear, disgust) within the Self, and suggest the need for ecological interventions that address beliefs about the Self in context.

## Methods

### Data

This paper will present novel analyses conducted on partially published datasets [32, 33]. We employed a mixed-method convergent triangulation design [42] to explore representations of mental illness of health and illness [32, 33]. The study involved three forms of data: news reports (*N* = 529), small focus groups (*N* = 20), and one-to-one interviews (*N* = 19) (Tables 1 and 2).

**Table 1:** Newspaper frequency statistics

Media Outlet	Frequency
The Guardian/Observer	119
Google News	93
Google Search	92
Daily Mail	54
LADBible	41
BBC	37
Youtube News	24
ITV	23
Buzzfeed	19
Sky News	18
Channel 4 News	9

Reprinted from “Charting an Alternative Course for Mental Health-Related Anti-Stigma Social and Behaviour Change Programmes” by D. Walsh and J. Foster in IJERPH , 19(10,618) , p.5 , Copyright 2022 by the authors [43]

**Table 2:** Participant demographics

		Interviews (*N* = 19)	Focus Groups (*N* = 20)
**Gender**	Male	7	3
	Female	11	17
**Age**	Mean	22	23
	Range	18–30	18–35
**Qualification**	Undergraduate	13	10
	Postgraduate	5	10
**Subject**	Psychological and Health Sciences	7	10
	Social Sciences and Humanities	6	7
	Natural Sciences	5	3
**Citizenship**	UK	11	14
	EU	7	6

Note: Charting an Alternative Course for Mental Health-Related Anti-Stigma Social and Behaviour Change Programmes” by D. Walsh and J. Foster in IJERPH, 19(10,618), p.6, Copyright 2022 by the authors [43]

We will now briefly summarise materials and methods to support comprehension. To limit data recycling and focus on the novel contributions themata might make to mental health-related anti-stigma efforts, please see [33] for a full account of the construction of datasets. All interview (LRS-18/19-9068) and focus group (LRS-19/20-14053) participants gave written informed consent, and the study was approved by the King’s College London college Ethics Committee.

#### News reports

We used three groups of search terms to develop the corpora. They included ‘mental’, ‘depression’, or ‘schizophrenia’; ‘student’, ‘university’, or ‘college’; and ‘housing’, ‘accommodation’, or ‘flat’. The search terms were written in English and covered publications from 01/01/2010 to 01/01/2020. A corpus of 529 news reports was compiled. The selection of news outlets was determined by the 10 news brands regularly self-reported, via any platform, as read by 16-24-year-olds in the UK, and included a range of the political spectrum, broadsheets versus tabloids (e.g., The Guardian for left-wing readership and broadsheet, and the Daily Mail for right-wing readership and tabloid). The corpora of news reports was rich and multi-media, encompassing news reports with both text and imagery [32, 33].

#### Interviews & focus groups

All interviews and focus groups were conducted between 2019 and 2020, and interview questions have been published previously in [33]. Transcriptions of focus group discussions and interviews were produced verbatim [32, 33]. All datasets concerned students’ representations of mental health and illness related to shared accommodation. We focused on students’ representations as they are considered an important group to target on account of their increased likelihood of personal and close contact with experiences of psychological distress [3, 44] and focusing intimate forms of contact commonly provide insight into the latent forms of lay representation [19, 40]. All students self-reported having no history of mental illness, as previous studies suggest that experiences of mental illness differentiate representation [45], although we found expressions of psychological distress were common in the sample [32, 33]. Students studying psychological and health sciences compromised roughly half the sample (*N* = 17/39).

### Analytic procedures

#### Clustering & network analysis

We duplicated the three datasets and applied standard data processing techniques in TextEditor for compatibility with Python3 [32, 33]. We analyzed the text from each dataset as a network of related words using a clustering coefficient (CC) algorithm as part of NLP. This algorithm measures how often and how close words appear together [33, 46]. The CC scores help us understand what concepts contained in the text. By looking at these scores alongside direct quotes, we can see how meanings gather around key concepts [33, 47]. For each dataset, we ranked the top 10 CC scores and provided illustrative direct quotes for each ranked word, following the conventions of mixed-methods research [33].

#### Thematic analysis

Each corpus was independently subjected to themata analysis supported by natural processing. Themata analysis was not solely the product of natural language processing but enriched by it. Specifically, words indicated by the clustering were used to keyword search the texts and support the researcher’s exploration into the themata utilised in sense making.

Identifying themata was a progressive, iterative process of open, axial, and selective coding. Open coding involved a thorough reading of the text. The open codes generated encompassed both semantics and pragmatics. Specifically, we paid close attention to the use of quantifiers (e.g., all, many) and logical connectives (e.g., not, if) used to represent mental illness, along with symbols (‘lock’ as protection), metaphors (e.g., ‘spiral’ as degeneration), and affects (e.g., fear, unease). We also focused on how language was used in context, coding elements that were strongly implied but not explicitly vocalized, potentially reflecting concerns about social desirability. These codes were generated through the authors’ close manual reading of the text, and through a keyword-in-context approach, indicated by words with high cluster coefficient scores (e.g., contagion). The initial open codes were then subjected to axial coding, where they were grouped into working categories and sub-categories. Finally, selective coding was applied to identify the themata or dialogical units. While this was a progressive process, it was particularly during selective coding that selective absences were commonly realized (e.g., recovery), often necessitating further open coding. After selective coding was complete, an uncoded sample was reviewed using the coding book to ensure consistency. It is in this final stage that is central to the differentiation between producing themata rather than themes. Whereas both involve clustering qualitative data, the identification of themes is concerned with repeated patterns, often interpreted as beliefs or opinions [47]. In contrast, the relational quality of themata focuses on the possible tensions in meaning-making, enabling the description of both resistance and reproduction of stigma through the same thema.

#### Triangulation

The themata analysis outcomes, bolstered by natural language processing, included three thematic coding books and three tables of cluster coefficient rank scores. We carefully reviewed the findings, paying special attention to the mixed feelings and opinions expressed in each situation and noting any differences between various situations. We refrained from imposing a rigid structure on the data. Instead, we critically assessed the ambivalence of understandings conveyed through the themata, explaining potential ambiguities by considering the role these understandings played within their communicative contexts. Our confidence in the findings was reinforced through triangulation of qualitative and quantitative data and by adopting a dialogical method to establish validity [42, 48].

## Results

### Summary

First, we will briefly summarize the results to help with understanding, which will be detailed later. Participants understood mental health and illness through a main thema of Self/Other. This main theme influenced five sub-themata: normal/abnormal, harm/non-harm, bounded/non-bounded, moral/immoral, and permanent/impermanent. These sub-themata helped shape people’s feelings and opinions on mental health and illness, reinforcing social identities. Understanding was often unclear and incomplete, based on symbols and images rather than solid knowledge. Tables 1, 2 and 3 show the top 10 keywords with a direct quote for each from the data. These keywords and quotes help us highlight potential meanings in the text, which will be discussed in terms of themes in the rest of this Results section [33].

**Table 3:** Table of cluster coefficient rank scores – interview corpus

Interview	Illustrative Quote
Lack	*“poor mental health. I guess more towards a lack of one’s own emotions*,* control emotionally*,* or thoughts as well.”* (IP10)
Worry	“you’d *probably worry about the cupboards and the cabinets with drugs inside even if it is just over-the-counter paracetamol*.” (IP13)
Fear	*“something like fear. You might discover some like you don’t know about*,* some negative things like—like you don’t want to admit you have like this negative trait in you.”* (IP3)
Separate	*“you can’t take part in like social life in what is considered to be like a normal way of being on everyday life. You have to kind of like*,* you’re separated from society when you have a mental illness. It’s sort of*,* there’s something about*,* you know*,* like your abnormal sort of in your—”* (IP12)
Fair	*“It’s about being firm but fair. In a sense*,* you have to have barriers to who you interact with and who you let know you let know your personal stuff*.” (IP5)
Hours	*“Every day*,* 8 am to 8 pm… That’s 12 h in a day. I think there’s a week or something… after some time you’re not even sure the difference between yourself and like—”* (IP7)
Lives	*“people who have very serious things that affect not only their day-to-day lives but their health*,* and their physical health*,* their interaction with other people and serious cases of bipolar and depression can lock you away from others”* (IP8)
Scare	*“You want to be distant from them somehow… That idea of being*,* “I’m kind of scared of you.”* (IP15)
Cope	*“literally withdrew from society in a way*,* so she says she just felt she can’t cope with day-to-day life.”* (IP4)
Reality	*“you would feel like you’re being away from reality*,* away from–I don’t know.”* (IP17)

### Self/other thema

A Self/Other theme was fundamental in maintaining mental health-related stigma in society. Through this theme, participants reinforced the idea that mental illness is different and separate from themselves. This perspective limited their full understanding of mental illness in several ways. A common approach was to view people with mental illness as part of an out-group, blaming them for their marginalization.

*“They want to be closed up*,* but deep down*,* they’re just unhappy with themselves. That’s why they’re closing themselves up*,* I think. For me*,* I’m happy in myself*,* I’m confident… I choose to be closed off. They choose to be as well*,* but like– I don’t know how to describe it. I’m happy that I’m closed off.” (*IP1).

IP1 represented mental illness as a negative form of personhood valanced against positive Self-attributions. IP1 fluidly uses words such as ‘they’ and ‘them’ to convey their perception that individuals with experiences of mental illness choose separation and sadness. Rather than representation being external or primary to personal experience, through the Self, people engaged in a motivated representation of mental illness as Other or ‘not-me’.

The relations between Self and Other were partially phenomenological. For example, in table one, we can identify expressions of ‘fear’ and ‘worry’ and even ‘dread’ and ‘despair’. Similarly, in the interviews, expressions of fear were common (Table 3). Whilst fear was also referenced in the focus groups (Table 4), its expression was less pronounced, and participants were oriented towards feelings of frustration or moral anger.

**Table 4:** Table of cluster coefficient rank scores – focus group corpus

Focus Group	Illustrative Quote
Perfect	“someone who looks and acts perfectly fine to you and you don’t notice anything when they—” (FG4P1)
Permanence	“*I suppose it’s this association of*,* um*,* permanence and of severity in how much it affects you.”* (FG3P3)
Pressure	“Let’s *say another roommate puts that pressure on you and doesn’t care about any other responsibilities or whatever.”* (FG3P1)
Responsible	“*You can get to a point where you feel like you’re responsible for that person… And like*,* that could– Because obviously*,* you were not like trained to deal who and whatever they have*,* so that can have a like a really more negative effect on you than you realise.*” (FG4P2)
Interpret	*“interpret it in a different way. Yeah*,* there’s a lot of environmental triggers like you know*,* you– media and all that stuff.”* (FG3P2)
Sick	*“kind of get sick of them. And they’re saying*,* look*,* I wish I didn’t have to spend time with this person because it feels like a big strain.”* (FG3P1)
Strain	*“If someone has mental health problems*,* then that might be a strain or a burden on those relations”.* (FG3P3)
Suicide	*“I think the first one we kind of think about it’s like depression and like suicide-related attitude.”* (FG2P1)
Trigger	*“seeing them with a weapon would look kind of trigger a fear response.”* (FG1P1)
Overwhelmed	*“someone who’s got mental health*,* I feel like they may feel more overly well-well–more overwhelmed than like someone who’s healthy.”* (FG5P1)

In addition, we found an emphasis on Otherness in perception. For example, in the media, the language was of ‘discovery’, in which the public ‘dreaded’ finding more ‘dead’ students (Table 5). Conversely, the conceived threat posed by mental illness was its perceived imperceptibility. FG4P1 (Table 4) leaves open-ended the statement: “*you don’t notice anything when they—”*. Still, and in often subtle ways, representations of the Other were as much about the Self as they were about understanding mental illness. Whilst it was very much in evidence that people feared the Other, mental illness was also feared for what it might reveal about the Self. For example, IP3 explains: “*fear. You might discover some like you don’t know about*,* some negative things like—like you don’t want to admit you have like this negative trait in you*” (Table 3).

**Table 5:** Table of cluster coefficient rank scores – media corpus

Media	Illustrative Quote
Contagious	*“she felt isolated when her friends were judgemental towards her*,* while her teachers would not take her back to school because they were worried about ‘contagion.’”* (BBC, 29.11.2016)
Inquest	*“Much loved Aston University student was found dead in halls of residence Coroner said she could not be sure final year languages student Sam Croydon intended to kill himself or if it was a cry for help.”* (Birmingham Live via Google Search, 04.02.2019)
Coroner	*“That sense of dread is known across the Kimberley. In February this year*,* coroner Ros Fogliani released her report into the deaths of 13 Aboriginal children and young people who died in the Kimberley between 2012 and 2016.”* (Buzzfeed [Australia], 14.06.2019)
Market	*“The current generation of undergraduates could be under greater pressure than previous generations because of increased study costs and an increasingly competitive jobs market.”* (BBC, 04.09.2017)
Counselling	*“More than a quarter (26%) of those do not get treatment*,* and only one in 10 use counselling services provided by their university…it is hard to deny that university is*,* in many ways*,* a petri dish of potential suffering.”* (The Guardian, 29.10.2015)
Rent	*“An infestation of rodents. Does this sound like a nightmare of your student days? Well*,* you’re not alone: a new survey reveals that these are the most common complaints for student accommodation – even as the cost of rent is going up.”* (Huffington Post UK via Google News, 13.03.2019)
Stigma	*“At-risk students waiting MONTHS for therapy as the demand for mental health care surges at seven times the rate of student body growth… experts attribute the rise to a fading stigma*,* social media fuelling anxiety and worry in the wake of mass shooting.”* (The Guardian, 16.09.2019)
Development	“*City Gate development in the Castlefield area of Manchester*,* which is built with combustible cladding. My mental health is at breaking point*,*” said Katie Peate*,* 28*,* who bought her flat there for £220*,*000 and now fears it could burn down at any time.”* (The Guardian, 25.04.2019)
Statement	*“Laughing schizophrenic cut off flatmate’s head after killing him with a cleaver*,* court hears. The family of Mr Marquez wept in court as they heard the details of their loved one’s death. In statement after the hearing*,* his mother Maria Carmen Marquez Torres*,* said: “Sergio’s life was cut short*,* a boy that only wanted to work*,* learn*,* help others*,* enjoy the life that God gave him and make everybody that knew him happy.”* (The evening standard via Google News, 03.12.2013)
Property	*“the property began to smell like “there was something dead” inside it.* (Liverpool Echo via Google News, 26.03.2019)

Rather than a fundamental lack of knowledge about mental illness, we found that comprehension of mental illness through contact was perceived as risky. This is highlighted by IP7 (see Table 3). While there was clear evidence of fear stemming from the unfamiliarity with mental illness, IP7 was also concerned about the implications of symbolically sharing space with individuals who have mental illness experiences. Sharing space was seen as a threat to their own uniqueness, making students want to keep a clear separation between themselves and the perceived Otherness of mental illness. Specifically, understanding mental illness through contact generated discomfort in individuals, which in turn motivated the perpetuation of a representation of mental illness as ‘Other.’

### Normal/abnormal thema

An important way of knowing was through comparison between perceived normal and abnormal behaviour. Yet, as mental illness was believed to relate to differences in daily living and causes beyond in-group perception (e.g., contagion (Table 5)) and to have a broad range of symptoms and signs partially shared with ‘normal’ psychological distress, ambiguities were pervasive in a normal/abnormal thema. However, at the level of representation, ambiguity was not a source of tension. Instead, through beliefs of alterity, participants used mental illness as an identity marker to distinguish normal and abnormal behaviour.

How alterity related to a normal/abnormal thema varied between communicative contexts. In focus groups, students particularised understandings to affirm mental illness’ associations with abnormality. For example, FG3P2 used the word ‘interpret’ (Table 4) to explain how a multitude of factors, such as contact with the unknown (*“you meet someone you don’t know”*), can ‘trigger’ persistent abnormal processes in the Self. Similarly, FG5P1 contends that groups with ‘mental health’ may be more inclined to feel ‘overwhelmed’ than ‘healthy’ people (Table 4). Abnormality was also implied through symbolic imagery. The image of a ‘petri-dish’ and ‘growth’ (Table 5) were common in constructions of mental illness, and normality was represented in contrast to a nightmarish ‘infestation’ of ‘rodents’ (Table 5); imagery commonly used to Other groups associated with socially undesirable illnesses (Sontag, 1978).

In the personal context of a one-to-one interview, students explained how the perceived abnormalities associated with mental illness differed from their personal daily experiences. IP10 imagined ‘poor mental health’ as personally experienceable ‘simple’ and ‘negative’ ‘day-to-day’ situations (Table 3). Yet, their understandings of how they, or those like them, would experience these situations were related to perceived Otherness of mental illness. Namely, a ‘lack’ of ‘emotional’ self-control, or the orientation towards a ‘pessimistic’ type of perception, rendered the experience of common situations different for those with a ‘strong’ mental health compared to those with ‘poor mental health’ (Table 3).

In summary, Participants understood mental illness through comparisons of perceived normal and abnormal experiences. This understanding varied by context, with focus groups emphasizing abnormality through symbolic imagery and personal interviews highlighting differences in daily experiences based on perceptions of emotional control and outlook. We will now see that the practices of dividing ‘normal’ and ‘abnormal’ experiences were linked to perceptions of harm.

### Harm/non-harm

The strong association of mental illness with harm instinctively reinforced negative perceptions of group differences. Harms were broadly defined and included fears of contagion, social isolation, physical violence, and undue burden, depending on the context of communication.

In the media, repeated sensationalised stories of death in student accommodation firmly associated mental illness with incomprehensible harm. Contagious, inquest, and coroner had the top-three CC scores (Table 5). For example, in Birmingham Live (Table 5), through the ‘inquest’, the author ‘revealed’ to the public what occurred at the ‘hall of residence’. However, reaffirming a representation of mental illness as uncertain and unknown, the author highlights how the *“coroner said she could not be sure … Sam Croydon intended to kill himself or if it was a cry for help”* (Table 5). Similarly, a reporter described how *“that sense of dread is known across the Kimberley”* (Table 5), suggesting that these ‘unknown’ harms are already developed in representation.

Students described multiple personal experiences of psychological distress to associate mental illness with a loss of Self. In focus groups, students especially focused on the perceived risks of undue dependency. For example, in Focus Group 3 (Table 4), ‘spending time’ with someone with a mental illness was represented as a ‘strain’ or ‘burden’ on the Self, and willingness for intergroup contact was conditional upon *“how much it affects you”* (Table 4). IP4 described how what is harmful about depression is that it ‘withdraws’ you ‘from society’ and ‘day-to-day life’ (Table 3). Similarly, although retaining distance from perceived Otherness of mental illness, students commonly referenced their own experiences of psychological distress and social isolation.

*“For a while now*,* when I’m with my friends*,* and I’m having fun*,* I just randomly feel guilty for no reason. I just feel like I just couldn’t fully put all my energy into it. I just felt so far away from everybody in that sense… you’re just feeling really empty inside.” (IP5)*.

For IP5, what was considered harmful about mental ill-health is it ‘isolates’ the self from one’s in-group. This was experienced in terms of ‘emptiness’, loss of ‘energy’, and ‘random’ or reasonless feelings of ‘guilt’.

These perceptions of harm and dependency illustrate the complex ways in which students navigate their understanding of mental illness and its impact on social relationships. We will now describe how students manage concerns for these harms by placing boundaries on intimate forms of contact.

### Bounded/non-bounded

Participants ambiguously used ‘boundaries’ to associate intergroup contact with risk. Rather than contact solely relating to a material space, contact was also located in in-group perception and was related to using symbolic language and social identity.

Contact taboos held a Self-protective function. IP3 also used the symbol ‘lock’ to describe how if they were to live with someone with a mental illness, they would ‘lock’ away personal spaces: “*Somebody with like crazy hair*,* like sore red-eye*,* and probably locked up in a hospital or someplace*“(IP3). They also used ‘lock’ to describe how mental illness was distanced to ‘strange’ spaces. At the same time, people believed that risks like contagion and social devaluation were not fully protected against through boundaries and separation. In their account of ‘contagion’ (Table 5), the BBC discusses how ‘Emma’ on account of her ‘bipolar disorder’, ‘self-harm’ and ‘suicide’ was removed from school. However, when Emma tried to go back to school, she was isolated by teachers and friends by ‘judgements’ and their fear of ‘contagion’.

Concern for contagion likely limited the formation of intergroup trust. Whilst students commonly discussed contact experiences, rather than contact constituting a consistent change mechanism, as contact was associated with risk and harm, through contact student reaffirmed Otherness and maintained social distance.

*“Someone’s mood can affect yours and like your mental health. It could end up*,* yes*,* I don’t know*,* just bringing you down as well*,* like by default.”* (IP14).

*“I can’t be around that for too long because it starts to affect me*,* and everything’s all good if it doesn’t affect me*,* but it once it does*,* I’m like*, [laughs] *‘Bye.’”* (FG4P1).

These examples suggest that rather than contact displacing public understanding, through contact people maintain understandings of mental health and illness, including historically rooted contact taboos, such as contagion beliefs [19, 44, 49–51], and canonic concerns for in-group safety were likely reimagined in the modern vernacular of ‘mental health’ or ‘mood’ [40].

### Moral/immoral

Self-Other dialogues reproduced a taken-for-granted belief that contact with mental illness challenges in-group social identity. We will now describe how representations of the Other degenerated the moral life of individuals with experiences of mental illness.

In the focus groups, a moral/immoral thema was implicitly communicated through expressions of discomfort and moral anger, along with discussions of fairness, responsibility, and burden. Intergroup contact was represented as unfairly bringing the Self into contact with the conceived Otherness of mental illness. FG4P2 explains that contact risks feeling unduly *“responsible for that person … which can have a like a really more negative effect on you than you realise”* (Table 4). Similarly, FG3P1 explained how students have a normative desire to not *“spend time with this person because it feels like a big strain”* (Table 4). FG3P1 also described how they *“didn’t ask for that… responsibility”* (Table 4) and blames individuals with experiences of mental illness for being a ‘burden’.

A review of media and interview CC rank scores (Tables 1, 2 and 3) suggested that mental illness was associated with something being ‘wrong’ or ‘bad’, especially when they felt mental illness was ‘too close’. Our close examination of the texts suggested that students held differentiated representations of social distance and morality. For example, IP11 distinguishes moral responsibilities based on felt closeness, showing that contact with others follows existing kinship and friendship patterns rather than being a single mechanism for change.

*She’s not my close friend… She’s not my boyfriend*,* or my sister*,* or anything. So boundaries it’s just sometimes I tell her I need space*,* and she just listened for that moment but won’t take it in—She understands for the moment but will do the same later.” (IP11)*.

IP11 experienced living with a friend with depression as a loss of power. Having their boundaries transgressed made them feel angry and constrained. Furthermore, moral involved complex representations of social distance. Unlike a ‘boyfriend’ or ‘sister’, IP11 was not ‘close’ to the friend in an emotional sense of the word and so felt they didn’t have responsibility for her welfare. Yet, the fact that the friend was perceived to transgress IP11’s ‘boundaries’ and entered IP11’s ‘space’ meant that the friend was likely materially close.

### Permanent/non-permanent

A discourse of permanence was largely developed through media through emotionally compelling accounts of violence perpetrated by individuals with experiences of mental illness against perceived vulnerable groups:

*“The 20-year-old university student … was in shock as to how the only person she’s ever loved would do that to her. The effect on her was severe and the trauma likely to be lifelong.”* (Cornwall Live via Google News, 15.11.2019).

In the public’s imagination, these articles likely reinforce the belief that contact with mental illness carries the risk of extreme violence and ‘life-long’ negative impacts on one’s mental health. This is not to downplay the necessity for criminal justice. Rather, it aims to emphasize how a selective and disproportionate emphasis on depicting mental illness through the lens of criminal proceedings likely perpetuates a belief among the public of assumed permanent risks associated with such contact.

The media’s portrayal of mental illness as a permanent risk is likely mirrored in the notable paucity of discussions about recovery in the interviews and focus groups. Permanence, as a theme, is only once explicitly indicated in the CC rank scores tables (seven). FG3P3 stated, *“there’s nothing that they can change … it’s this association of*,* um*,* permanence and of severity in how much it affects you.”*. We found a taken-for-granted belief that mental illness has permanent negative effects on the Self.

Beliefs of permanence were instead implied when students compared between perceived category groups:

*“I guess the implication of the word like illness being used is a bit harsher*,* like someone could have both like mental health problems and a mental health illness. But I guess like a mental problem does seem like*,* just like a symptom*,* like a thing that’s wrong that’s like even more easily solved. I feel like mental illness is kind of more like chronic or like diagnosed.”* (FG1P2).

In this example, FG1P2 compares ‘mental health problems’ and ‘mental illness’ to express a belief that issues which are not ‘chronic’ or ‘diagnosed’ feel less ‘harsh’. This comparison subtly suggests that the impossibility of recovery is not explicitly mentioned but is implied in representation. FG1P2 stated, ‘even more easily solved’. While this statement does not fully capture recovery as living to one’s ‘full human potential’ [10], it implies that achieving recovery is seen as relatively more attainable for individuals without a ‘mental illness’.

A Self/Other themata likely underpinned these seemingly neutral comparisons. Students commonly Othered mental illness to distinguish it from their personal experiences of distress.

*“I know from personal experience*,* I’ve gone through periods where*,* weeks at time capping in up to after two months*,* I can feel like utter trash*,* and it starts to impact my daily life… But this is something that’s being caused by something.”* (IP8).

In the example above, IP8, whilst acknowledging that they have for ‘weeks at a time’ felt like ‘utter trash’, felt categorically different from mental illness. To do so, they represented themselves in terms of having poor mental health. However, what rendered themselves different to mental illness was their recovery and comprehensibility.

In summary, we found that the media often portrays mental illness through emotionally charged accounts of violence, reinforcing the belief that mental illness is associated with permanent, severe risks and negatively impacting the perception of recovery. This perception is reflected in the interviews and focus groups, where students differentiate between ‘mental health problems’ and ‘mental illness,’ implying that recovery is more achievable for less severe issues and that mental illness carries an assumed permanence.

## Discussion

Overall we found that concerns for a loss of Self sustained a representation of mental illness as Other or ‘not-me’ [32, 40]. Concerns for the Self also shaped meaning-making in sub-themata, such that the public largely assumed mental illness to be a permanent negative form of personhood, characterised by abnormality, harm, distance, and immorality [11, 18, 32, 40, 48, 52, 53], alternative to sustaining positive Self beliefs [15, 54]. Mental health-related stigma was in part sustained by latent communications [15, 27, 33, 55]. Unlike logical decision-making models, people’s understanding of mental illness wasn't about having complete knowledge or no understanding at all. Instead, it was naturally shaped by their social identity and their perceptions of how society is organized [15, 54]. The novel theoretical-methodological approach, centering on themata, underscored selective absences [27] and the differentiation between 'types' of mental illness [32, 40]. These ambiguities played a significant role in enabling people to resist acknowledging the similarities between comprehensible forms of public psychological distress and mental illness. Yet, the differences between the Self and the Other were insecure. The negative feelings related to contact probably stemmed from the public's hidden worries about their own risk of developing mental illness [15, 54].

Given the complexity of mental health and illness representations and the tenacity with which the public 'Others' mental illness, we foresee limited value in continued focus on refining abstract and generic educational models for change. Researchers have commonly responded to the limited effects of knowledge-based interventions by recommending alternative curriculums, such as exploring the benefits of continuum and/or social models of mental health and illness, and emphasizing recovery [3, 10, 56]. In the short term, these are likely an advance on the categorical and biomedical models that dominate anti-stigma efforts, especially in lower- and middle-income countries [1, 2, 16]. However, since stigma is so ingrained in the largely intuitive process of developing understanding and is linked to negative affects in the Self [32], there is little evidence to support the continuation of knowledge-based strategies for change. Moreover, students with more exposure to professional knowledge concerning mental illness, such as those studying psychological and health sciences, who comprised roughly half the sample, were no less stigmatizing than assumed in public health campaigns. Instead of lacking knowledge about mental illness, we found that these students creatively used contemporary mental health-related language (e.g., 'mood') to 'Other' mental illness, sometimes even drawing on professional terminology [32].

Similarly, our results caution against the current overall preference for social contact as a generic model for change [1, 7, 26]. While it was evident that knowledge-contact beliefs were central to the maintenance of mental health-related stigma, when contact was undesired or felt to be threatening, this motivated the reproduction of a representation of mental illness as 'Other'. While we did find conditional expressions of empathy, these were for individuals who already felt close (e.g., a sister), and overall representations of contact with individuals with experiences of mental illness were negative. Rather than contact being an ingredient for change, attention to themata suggests that people experience contact in such a way that affirms its perceived 'Otherness,' such as by associating mental illness with historically rooted concerns for contagion, violence, and moral degeneration [18, 44, 49], while also holding a self-protective generative function [54]. We found a Self/Other thema informed representations of contact; through contact, people reproduced a positive social identity as different and distant from individuals with experiences of mental illness, whilst also being cognizant of harm incurred when feeling isolated [57].

We acknowledge the desire to propose general recommendations to limit the unintended consequences of current educational models and contact-based approaches for anti-stigma change. However, given the diverse and ambivalent ways in which people maintain the ‘otherness’ of mental illness, we argue that themata provides an alternative methodological-theoretical framework to describe in context the latent and varied ways individuals relate to mental illness, and likely has the potential to elucidate the uncertain processes through which change is communicated to oneself and others. This would present a significant change in approach to mental health-related stigma. Rather than assuming mental health-related knowledge is caused by a lack or deficiency in knowledge, it opens the possibility for practitioners to be in sustained communication with the public, continually evaluating possible benefits and unintended consequences throughout the change process.

Instead, of proposing corrections to reduce the unintended consequences of anti-stigma programs, we propose that the researchers should engage with a thoroughly socio-ecological understanding of stigma and employ themata as a theoretical methodological-framework for describing the forms of understanding which groups maintain mental health-related stigma in context. By adopting an ecological approach, we move beyond the uni-directional limitations of previous campaigns. These campaigns predominantly focused on rectifying a perceived deficit in knowledge within a specific area, such as the misconception that mental illness is uncommon (and therefore abnormal). Instead, we acknowledge that the belief in the rarity of mental illness is closely linked to the motivated perception that mental illness is ‘Other’ and not related to oneself.

To do so practitioners may benefit from learning from aligned studies because there has been limited research into the policy implications of public mental health-related themata. Bertoldo & colleagues (2015) presents a pertinent case study. They conducted a longitudinal mixed-method research project to explore how groups developed representations of smart meters during a pilot project [58]. They found citizens embodied practices towards smart meters themselves, as well as sustainable consumption in general (e.g., public transportation in rural areas), and daily life events were organised through three themata: collective vs. individual (daily life); private (my behaviour) vs. public spheres (others’ behaviours); and consumption: individualist vs. collectivist [58]. Policymakers could propose ways to respond to current ecological changes and public beliefs and behaviours identified in earlier research [58]. By iteratively revealing lay themata in their complexity, policymakers could comprehend their citizen’s needs and proactively respond to implementation barriers before a full project rollout [58]. Moreover, identifying themata supported alternative ways of knowing, in particular, a reconsideration of sustainable consumption from being an individual issue to a societal one [58].

Exploring the themata through which policymakers themselves comprehend mental health and related stigma may provide a basis to align the ways policymakers comprehend the issue with how the lay public represents it [36, 58]. Moreover, themata may support sustainable behaviour change strategies, as themata focus attention on the historically enduring aspects of public understanding, such as representations of Otherness [33, 39, 41], and support solutions responsive to the ambiguities present in public understanding [39, 41], rather than developing solutions responsive to just one theme (e.g., responsibility in the first stage of the Time-to-Change Campaign). This could hopefully foreground communications which promote public trust that challenging mental health-related stigma does not challenge positive in-group forms of social identity [7], a possible innovation in challenging the motivations that sustain representations of Otherness and reproduce mental health-related stigma in society [1, 33]. Furthermore, we should highlight the direction of travel in British universities' approach to mental health and illness [59]. Whole-university approaches are being advanced in higher education systems, emphasizing the need to consider the university as a site for the reproduction of psychological distress beyond individual clinical symptoms [59]. Although, to our knowledge, none have specifically piloted themata as a means of stigma alleviation in the university context, these approaches are aligned in their emphasis on the need for socio-ecological approaches to mental health and illness and the centrality of communication processes [59].

## Conclusions

Anti-stigma social and behavioural change strategies would likely benefit from paying greater attention to public communication patterns and the vital role themata have in constraining and generating ways of knowing mental health and illness.

In this study, mental health and illness was represented in a way which sustained positive Self and in-group representations. We found public Self/Other dialogues to motivate and constrain comprehension, such that representations of mental illness were characterised by abnormality, harm, immorality, distance, and permanence. Our research also showed that these views can be inconsistent. Rather than expressing coherent explanations for the perceived differences between themselves and mental illness, we found people used ambiguity and absence of Other mental illnesses.

Unfortunately, our focus on latent aspects of communication found little to recommend continuing dominant change strategies. Indeed, the felt need to Other was likely instinctive [9, 10], and education and contact-based strategies suffer profound issues in problem conceptualisation [1]. We hope that our exploration into lay mental health-related themata will provide practitioners with an alternative basis for addressing public understanding and, in particular, support them in challenging the identity protective functions of stigma.

## Data Availability

No datasets were generated or analysed during the current study.
